# Department of Obstetrics and Gynecology

**Published:** 1893-09

**Authors:** 

**Affiliations:** F. R. C. S. Ed., Professor of Anatomy University of Texas; late Physician for Diseases of Women, Edinburgh Provident Dispensary; Galveston


For Texas Medical Journal.

### OBSTETRICS AND GYNECOLOGY.

By Wm. Keiller, F. R. C. S. Ed., Professor of Anatomy University of Texas; late Physician for Diseases of Women, Edinburgh Provident Dispensary.

---

COMBINED SYMPHYSIOTOMY AND CRANIOTOMY IN OSTEOMALACIAL PELVIS.—Dunmock reports a delivery (successful as regards the mother) by combined symphysiotomy and craniotomy in an extremely contracted osteomalacial pelvis. Patient pale, weak, emaciated; 4 feet 3 inches high; exhausted by prolonged labor. Pelvis rostrated. Iliac crests 8½ inches apart, spines 8 inches, intertrochanteric 8½ inches, external conjugate 6 inches. Patient too exhausted for Cæsarean section. The symphisis, after delivery, was united by deep wire sutures passed through the periosteum and ligaments, the bones being too soft to admit of drilling. Patient recovered well, the symphysis uniting firmly. —*Brit. Med. Journal*, June 24, 1893.

---

THE ABSORPTION OF FIBROID TUMORS OF THE UTERUS.—Alban Doran reports a case. Patient, aged 40; seen May 9, 1890. Complaining of pain and dysuria, caused by a blow on a uterine fibroid, which had been felt to exist for three years. The tumor filled the left iliac fossa and reached higher than the umbilicus. It was subserous and corporeal. Os uteri close to pubes. Relieved by rest. On February, 1891, patient again seen. There had been a fœtid discharge in August, 1890. The tumor now could only be felt just above the pelvic brim. On November 25, 1892, there was no trace of the tumor and the uterine cavity measured 3½ inches. Uterus freely moveable. Doran had collected reports of thirty-seven cases, many of them of complete disappearance, before the menopause. Thirteen of these were associated with pregnancy, six with inflammatory conditions, ten not so associated.

Climacteric disappearance does not commence until two years after the menopause. Duncan and Hewitt also reported cases of disappearance before the menopause. Duncan had opened the abdomen in his case for a bleeding fibroid reaching to the umbilicus, but extensive adhesions caused the operations to be abandoned; 1½ years afterwards the tumor had disappeared.—*Brit. Med. Journal*, June 24, 1893.

---

RUPTURE OF THE UTERUS—PACKING WITH IODOFORM GAUZE—RECOVERY.—Herzfeld reports two cases. First patient found collapsed—right hand presenting—feet to the right. Decapitation, delivery. Laceration in cervix, extending into peritoneum, probably caused by attempt at turning; little hemorrhage; no signs of sepsis; plugged with iodoform gauze; recovery.

Second case, primipari, aged 22; attempts had been made to apply forceps. On admission patient bleeding freely; piece of bruised tissue hanging from vulva. Rent from vestibule through lateral wall of vagina into pelvic cellular tissue and peritoneum. Child delivered by axis traction forceps and rent plugged. Recovery —*Cent. f. Gynæk.*, No. 17.

---

CÆSAREAN SECTION.—Skene Keith, speaking of Cæsarean section, considers it much safer for the patient than Porro's operation (removal of the pregnant uterus). With a conjugate above 2⅝ or 2¾, delivery by symphysiotomy is possible— below this, Cæsarean section and ligature of the tubes to prevent recurrence of pregnancy is the proper treatment where the child is to be saved. The operation is one of the simplest in abdominal surgery. Cleanliness and rapidity in operating are the essentials to success.—*Brit. Med. Journal*, July 1, 1893.

---

DEATH FROM LEAD POISONING DUE TO THE USE OF "DIACHYLON" PLASTER (LEAD PLASTER) TO PRODUCE ABORTION. —Pope reports two cases of death from lead poisoning, where careful enquiry elicited that a popular superstition exists that "diachylon" taken internally will induce abortion, and the patients had been in the habit of taking diachylon, rolled into pills, with that intent. These two cases occurred within two years in Leicester, but the use of diachylon for such a purpose may be practiced elsewhere.—*Brit. Med. Journal*, July 1, 1893.

---

HEREDITARY TENDENCY OF PLURAL BIRTHS.—Mari e quotes a case tending to show that the male may inherit the faculty of begetting twins.

Two brothers belong to a family of sixteen, of whom six were twins. Each of these brothers, themselves twins, begot twins. A girl of the family presents a supernumerary nipple which has appeared in the family for four generations.—*Sem. Med.*, June 4, 1893.

---

STRANGULATED OBTURATOR HERNIA OF THE OVARY AND TUBE.—Von Rogner Gusenthal describes a case in a patient 66 years old. There were symptoms of strangulation, with pain and indistinct gurgling, but no distinct tumor, in the right groin; femoral hernia was diagnosed. On operation the crural canal was clear but a bulging was seen under the pectineus muscle. The muscle was divided and the sac of the hernia in a gangrenous condition, bulged forward. This contained the right ovary and tube and a coil of intestine, all gangrenous. The bowel was opened and three pints of fœtid fluid escaped through a rubber tube passed into it. The tube was changed, left in situ, and occasionally unchanged for escape of fæces or flatus. Patient died. The hernia had escaped through the obturator foramen.—*Wiener Med. Presse*, June 25, 1893.

